# Sustained release buprenorphine effectively attenuates postoperative hypersensitivity in an incisional pain model in neonatal rats (*Rattus norvegicus*)

**DOI:** 10.1371/journal.pone.0246213

**Published:** 2021-02-03

**Authors:** Alexandra Blaney, Katechan Jampachaisri, Monika K. Huss, Cholawat Pacharinsak

**Affiliations:** 1 Department of Comparative Medicine, Stanford University School of Medicine, Stanford, California, United States of America; 2 Department of Mathematics, Naresuan University, Phitsanulok, Thailand; University of Pittsburgh, UNITED STATES

## Abstract

Despite the need for safe and effective postoperative analgesia in neonates, research regarding pain management in neonatal rodents is relatively limited. Here, we investigate whether sustained release buprenorphine (Bup SR) effectively attenuates thermal hypersensitivity in a neonatal rat model of incisional pain. Male and female postnatal day 3 Sprague Dawley rat pups (n = 34) were randomly assigned to one of four treatment groups: 1) saline (control), 0.1 mL, once subcutaneously (SC); 2) buprenorphine HCl (Bup HCl), 0.05 mg/kg, once SC; 3) low dose Bup SR (low-SR), 0.5 mg/kg, once SC; 4) high dose Bup SR (high-SR), 1 mg/kg, once SC. Pups were anesthetized with sevoflurane and a 0.5-cm long skin incision was made over the left lateral thigh. The underlying muscle was dissected and closed using surgical glue. Thermal hypersensitivity testing was performed at 24 h prior to surgery and subsequently at 1, 4, 8, 24, and 48 h post-surgery using an infrared diode laser. Thermal hypersensitivity was attenuated at 1 h post-surgery in the Bup HCl group, while it was attenuated through the entire postoperative period in both low-SR and high-SR groups. This data suggests that a single dose of low-SR (0.5 mg/kg) or high-SR (1 mg/kg) effectively attenuates thermal hypersensitivity for at least 8 h in neonatal rat pups.

## Introduction

Evaluation and management of pain in neonates is an area of increasing focus within both human and veterinary medicine. Prior to 1980, it was common for human neonatal patients to undergo invasive surgical procedures without adjunctive pain medication [1]. The prevailing belief during this time was that the neonatal nervous system was not developed enough to feel pain [2]. Current research, however, indicates that neonates not only do experience pain, but that controlling pain early in life is critical to preventing lasting changes to developing somatosensory systems [3–5]. In human medicine, a number of analgesic agents have been evaluated in neonates and infants, including topical anesthesia, acetaminophen, and opioids [6]. Despite being widely used, both local analgesia with a topical EMLA cream and systemically administered acetaminophen failed to provide adequate pain control after common neonatal intensive care unit procedures [7,8]. Opioids are also commonly used in human neonates and have been shown to provide adequate procedural analgesia in this population [6].

In laboratory animal medicine, neonatal rodents are commonly used in a number of survival surgeries ranging from stereotactic [9] and neurological research [10] to cardiothoracic procedures [11,12]. Analgesia in neonates is complex and must take into account physiologic differences between neonates and adults. Rapid developmental changes in renal and hepatic function during the neonatal period affect the pharmacokinetics and pharmacodynamics of analgesic agents, often resulting in an initial reduction in dosing and clearance [13]. Neonates are also more sensitive to the adverse effects of many drugs, necessitating careful attention to technique and close monitoring after administration [14].

For the past several years, a sustained release formulation of buprenorphine (Bup SR) has been commercially available on the veterinary market [15]. It is an opioid analgesic that is often considered the ‘gold standard’ for postoperative pain management in adult laboratory animals [16]. Bup SR, like regular buprenorphine (Bup HCl), is a partial μ-opioid receptor agonist that has antagonistic effects at κ-opioid receptors [17] and is therefore preferred over other full μ-opioid receptor agonists, such as morphine, due to its wide margin of safety and longer duration of action [18]. Reducing the stress associated with handling and repeat injections is beneficial in all laboratory animals but could be especially useful when providing analgesia to neonates as disruptions to the nest and subsequent stress to the dam can predispose to cannibalization and maternal neglect [19]. A single dose of Bup SR is reported to provide postoperative analgesia for 48–72 h in adult rats [16,20]. It therefore represents a considerable refinement and improvement in animal welfare in veterinary postoperative care and has been validated in a number of adult rodent models [15,16,20,21]. Plasma concentration in rats administered Bup SR at both 0.9 mg/kg and 1.2 mg/kg remained within a range considered to provide analgesia 72 h after a single injection [21]. Although Bup SR has been widely used to provide longer postoperative analgesia in adult rats, its effects in rat pups is poorly understood.

To our knowledge, there are no published studies assessing Bup SR in neonatal animals of any species. Therefore, the aim of the current study was to examine postoperative analgesia using an incisional pain model of three-day-old rat pups given two different doses of Bup SR. We hypothesized that Bup SR given at both the low and high dose would safely and effectively attenuate thermal hypersensitivity for a longer duration than Bup HCl.

## Materials and methods

### Animals

One-day-old Sprague Dawley rat pups [(Crl: CD (SD) IGS), (*n* = 34), Charles River Laboratories, Hollister, CA] equal male and female, housed in litters of ten to twelve, arrived with their dam at the facility on day 0. Rats were free of rat coronavirus, rat Theiler virus, Kilham rat virus, rat parvovirus, Toolan H1 virus, rat minute virus, lymphocytic choriomeningitis virus, murine adenovirus types 1 and 2, reovirus type 3, Sendai virus, pneumonia virus of mice, *Mycoplasma pulmonis*, mites, lice and pinworms. Animals were housed in static microisolator cages on a 12:12 h dark:light cycle. Rat dams were fed a commercial diet (Teklad Global 18% Protein Rodent Diet 2018, Harlan Laboratories, Madison, WI) and were provided water filtered by reverse osmosis ad libitum. All experiments were approved by the Stanford Administration Panel for Laboratory Animal Care. All rats were treated in accordance with the *Guide for the Care and Use of Laboratory Animals* in a facility accredited by the Association for the Assessment and Accreditation of Laboratory Animal Care, International [22]. At the study’s conclusion, rat pups and dams were euthanized by carbon dioxide asphyxiation followed by decapitation as a secondary method of euthanasia.

### Experimental groups

Three-day-old pups of either sex were randomly assigned to one of four experimental treatment groups and the end observers were blinded to treatment group allocation. Treatment groups consisted of: 1) saline (Saline; 0.9% NaCl, Hospira, Lake Forest, IL) - 0.1 ml subcutaneous (SC) once (n = 9); 2) buprenorphine HCl (Bup HCl; Par Pharmaceutical, Chestnut Ridge, NY) - 0.05 mg/kg SC once (n = 8); 3) low dose sustained release buprenorphine (low-SR; Zoopharm, Fort Collins, CO)– 0.5 mg/kg SC once (n = 8); 4) high dose sustained release buprenorphine (high-SR) - 1 mg/kg SC once (n = 9). All injection sites were pinched for 5 seconds post-injection to prevent leakage.

### Anesthesia and surgical incision

Anesthesia was induced with an induction chamber (2L of 100% O_2_ with sevoflurane at 5%) and maintained with a nose cone mask (0.5L of 100% O_2_ with sevoflurane at 1–3%). The pups were placed in right lateral recumbency on a warm water circulating blanket set to 38°C (Stryker T/Pump, Portage, Michigan) for the entirety of anesthesia. After transfer to the nose cone, the experimental treatment was administered interscapularly. The thigh was surgically prepared with three alternating betadine (10% povidone-iodine, Purdue Products L.P., Stamford, CT) and 70% Isopropyl Alcohol USP (Henry Schein, Melville, NY) preparations. A 0.5-cm incision was made on the left lateral thigh. The underlying muscle was dissected, and the incision was closed with surgical tissue glue (VetBond, 3M, St Paul, MN). Pups were recovered in a recovery cage on a warm water circulating blanket and placed back to the dam once fully recovered.

### Thermal hypersensitivity

*Baseline thermal sensitivity*: At two-days of age (-24 h), laser stimulation was performed to determine baseline thermal latency. Skin temperature was measured prior to laser stimulation on the left and right lateral thigh using an infrared thermometer (Kintrex Infrared Thermomemter IRT0421, Kintrex, Vienna, VA). During stimulation and testing, pups were maintained on a warm-water circulating blanket. An infrared diode laser stimulator (LASMED, Mountain View, CA) was used at 490 mA with a cut off value of 19-seconds. The laser was focused perpendicular to the lateral thigh with 4 mm in diameter and 3.5 inches from the surface of the skin. Purposeful movement away from the laser was measured as thermal latency in seconds. Two measurements, taken 3 to 5 minutes apart, were averaged on the left thigh.

Thermal latency testing was performed at 1, 4, 8, 24, and 48 h post-surgery on the ipsilateral (left) thigh. Skin temperature was measured prior to each testing. Thermal hypersensitivity was defined as a significant decrease in withdrawal latency following focal thermal stimuli.

Pups were weighed daily, and incisions and injection sites were monitored daily for the duration of the study.

### Statistical analysis

Weight, sex, and thermal latency were analyzed with repeated measure with Bonferroni for multiple comparisons (SPSS, IBM, Somers, NY). Data are expressed as mean ± SEM. A *p*-value of less than 0.05 was considered as significant.

## Results

### Body weight and sex

**Fig 1:**
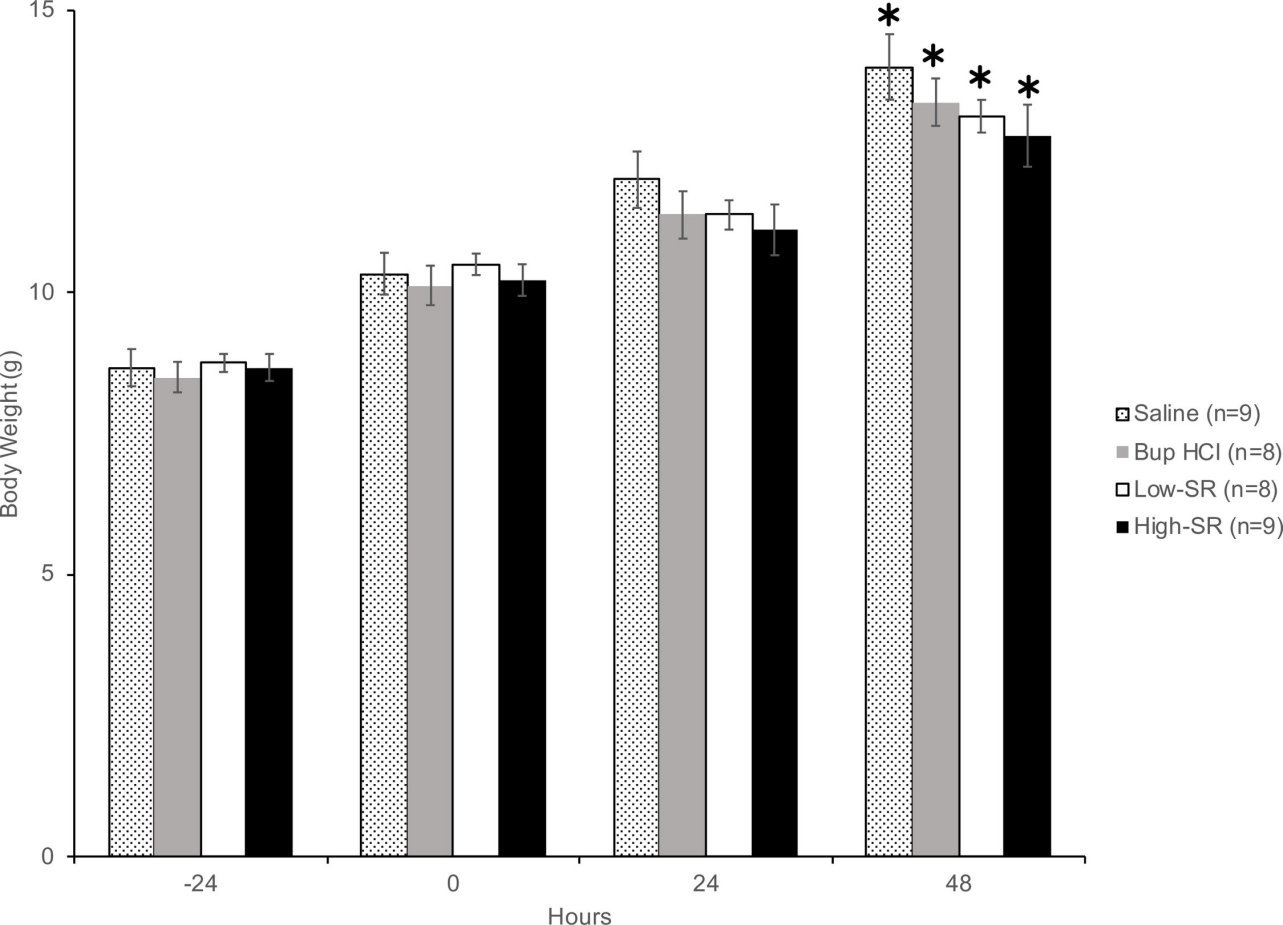
Body weights of rat pups throughout the study; mean ± SEM. *, 48 h weights were significantly (*P* < 0.05) higher than -24 h (baseline) weights for all treatment groups.

The weight of the rats in all treatment groups gradually increased from -24 h to 48 h of the experiment (Fig 1). Weight gain was statistically significant in all treatment groups from -24 h [Saline 8.7 ± 0.3 g, *F*(1,8) = 341.33, *p*< 0.001; Bup 8.5 ± 0.3 g, *F*(1,8) = 462.91, *p* < 0.001; Low-SR 8.8 ± 0.2 g, *F*(1,8) = 276.61, *p* < 0.001; High-SR 8.7 ± 0.2 g, *F*(1,8) = 136.90, *p* < 0.001] to 48 h (Saline 14.0 ± 0.6 g; Bup 13.4 ± 0.4 g; Low-SR 13.1 ± 0.3 g; High-SR 12.8 ± 0.6 g). Body weight did not differ between groups at -24 h, nor was weight gain different between groups over the course of the experiment. There were no sex differences for skin temperature [ipsilateral (*F*(1,26) = 0.019, *p* = 0.891) and contralateral (*F*(1,26) = 0.134, *p* = 0.718)] and thermal hypersensitivity test [ipsilateral (*F*(1,26) = 0.019, *p* = 0.892)].

### Skin temperature

**Fig 2:**
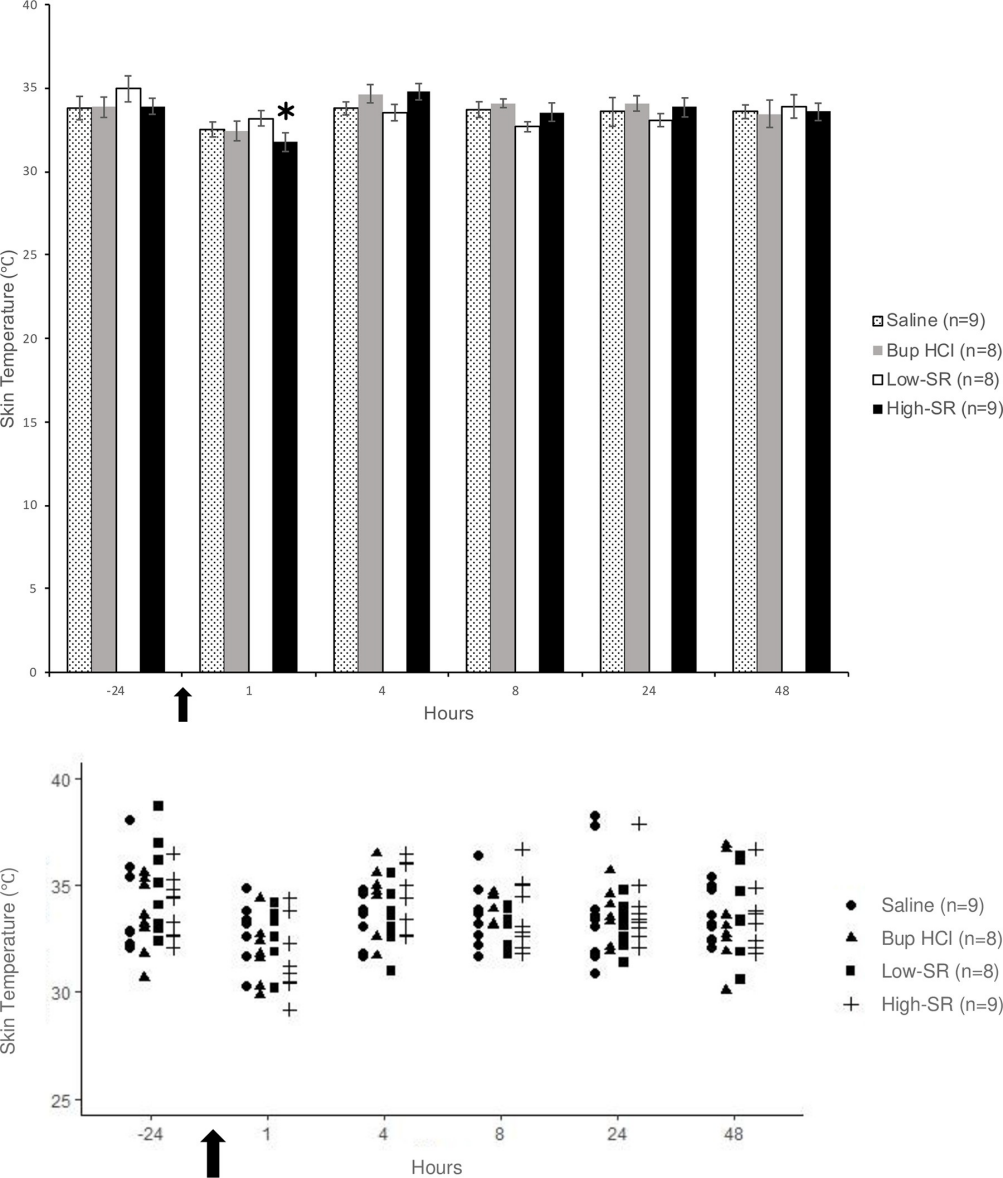
Skin temperature of the ipsilateral thigh measured in degrees Celsius (°C) shown as (A) mean ± SEM and (B) individual data points. *Values significantly (*P* < 0.05) different from the –24 h (baseline) value for the same treatment group. The arrow represents the time of surgical incision.

**Fig 3:**
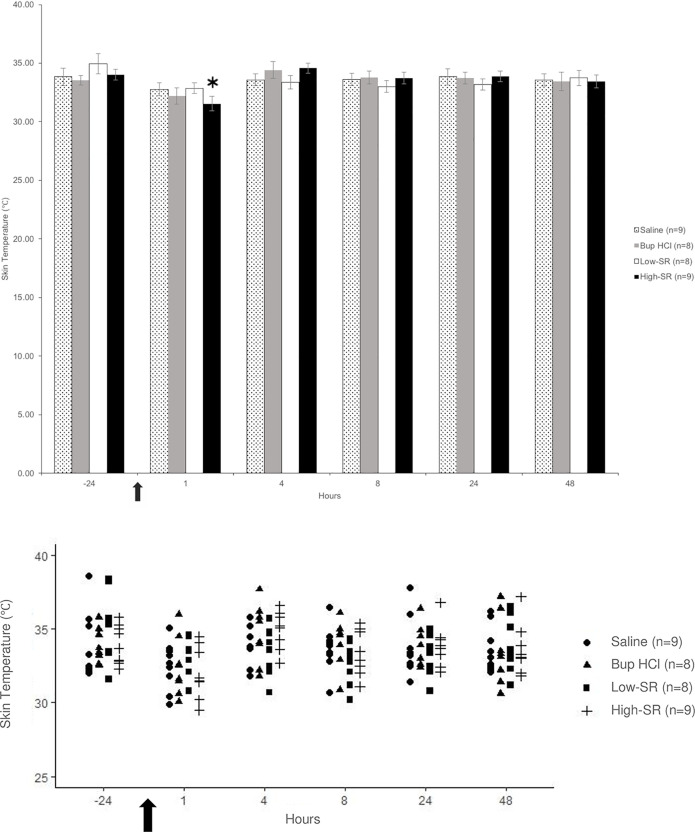
Skin temperature of the contralateral thigh measured in degrees Celsius (°C) shown as (A) mean ± SEM and (B) individual data points. *Values significantly (*P* < 0.05) different from the –24 h (baseline) value for the same treatment group. The arrow represents the time of surgical incision.

In the high-SR groups, the skin temperature was significantly lower in both the ipsilateral (31.6 ± 0.6°C; *F*(1,8) = 18.93, *p* = 0.002); Fig 2) and contralateral ((31.8 ± 0.6°C; *F*(1,8) = 17.91, *p* = 0.003); Fig 3) thighs at the 1 h time point as compared to -24 h (33.9 ± 0.5°C and 34.0 ± 0.4°C, respectively; baseline). There were no significantly different skin temperatures in any other groups at any time points.

### Thermal hypersensitivity

**Fig 4:**
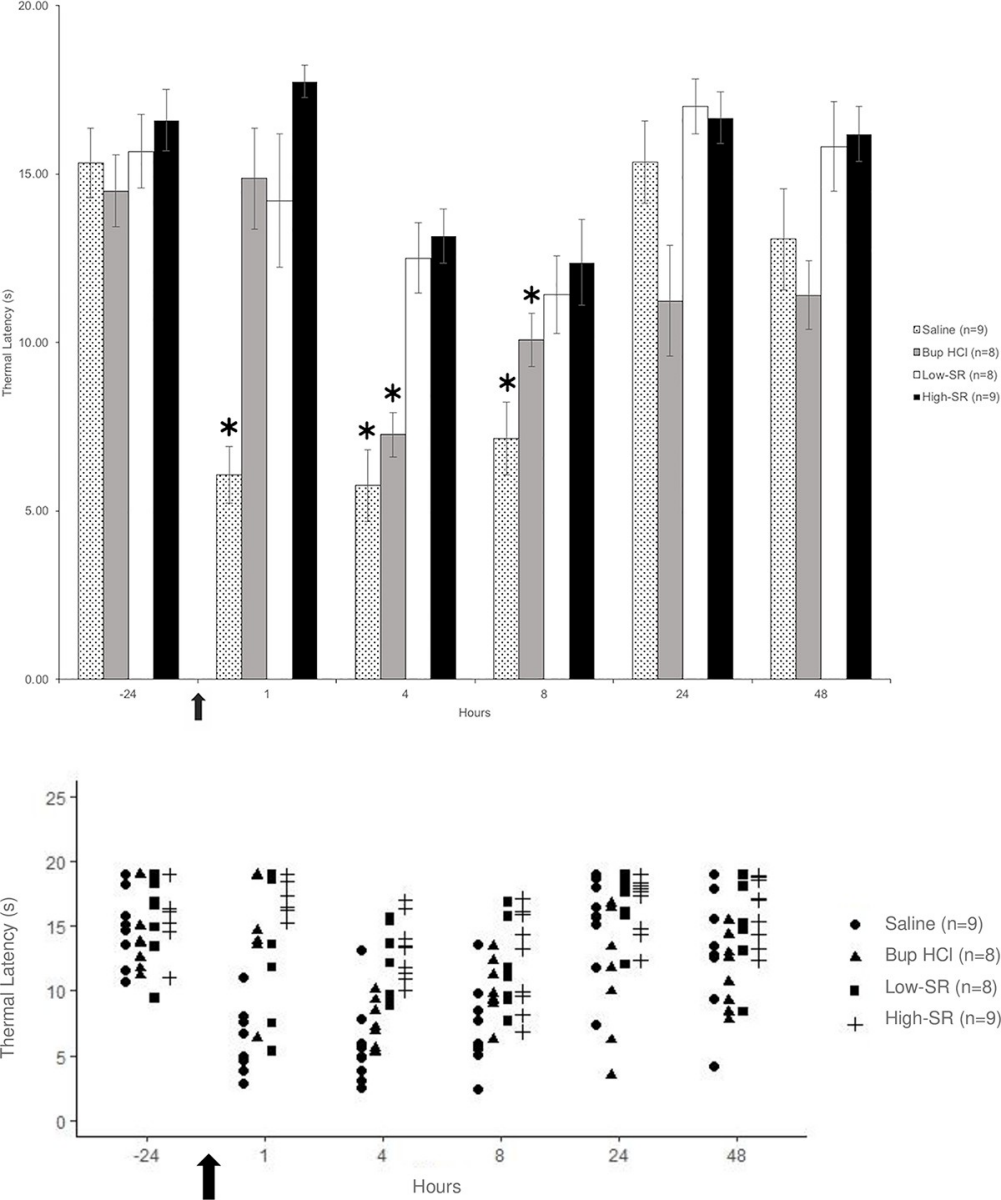
Thermal hypersensitivity (measured as thermal latency [s] to withdrawal) of the ipsilateral thigh shown as (A) mean ± SEM and (B) individual data points. *Values significantly (*P* < 0.05) different from the -24 h (baseline) value for the same treatment group. The arrow represents the time of surgical incision.

Thermal hypersensitivity did not differ between males and females at any time point. In the ipsilateral hindleg, thermal latency did not differ between groups before treatment (Fig 4). Rat pups that received saline had significantly reduced thermal latency at 1 [6.1 ± 0.8 s; *F*(1,8) = 42.98, *p* < 0.001], 4 [5.8 ± 1.1 s; *F*(1,8) = 61.77, *p* < 0.001], and 8 [7.2 ± 1.1 s; *F*(1,8) = 55.92, *p* < 0.001] h as compared with -24 h (15.3 ± 1.0 s; baseline). In the Bup HCl group, when compared with -24 h (14.5 ± 1.1 s; baseline), thermal latency was not different at 1 (14.9 ± 1.5 s), 24 (11.2 ± 1.6 s), and 48 (11.4 ± 1.0 s) h, while it was significantly reduced at 4 (7.3 ± 0.7 s), and 8 (10.1 ± 0.8 s) h. The thermal latency of rat pups in the low-SR and high-SR groups did not differ between any time points.

## Discussion

This is the first study demonstrating that a single dose of Bup SR (0.5 and 1 mg/kg) effectively attenuates thermal hypersensitivity for at least 8 h in a neonatal rat model of incisional pain. Thermal latency in this study was defined as time until purposeful movement away from the laser, and decreased latency correlated with thermal hyperalgesia. Withdrawal and behavioral response to noxious stimuli is seen in rat pups as early as postnatal day 1, and injection of irritating chemicals in rat pups at postnatal day 3 results in allodynia and hyperalgesia [23,24]. The behavioral responses of young pups to noxious stimulus include whole body wriggling as well as more localized withdrawal responses [14]. Thermal latency of neonates that received Bup HCl was significantly shorter at 4 and 8 h as compared to baseline, supporting our hypothesis that both low-SR and high-SR safely and effectively attenuate thermal hypersensitivity for a longer duration than Bup HCl in a neonatal rat model of incisional pain. Given that both doses of Bup SR had similar efficacy, we recommend the use of Bup SR at 0.5 mg/kg in neonatal rats for the management of postoperative incisional pain.

We chose to use a modified incisional model of acute pain because our lab has extensive experience with this model and has found it to reliably replicate minor postoperational pain. Previous studies utilizing this model showed that thermal hypersensitivity lasted at least 4 days when the hindpaw was incised [16,25–27]. In contrast to the original model [25], we incised over the lateral thigh rather than the plantar surface of the hindpaw due to the smaller size of neonates and the increased ease of performing thermal hypersensitivity testing over this location. An in-press study by our group found that a skin incision alone over the lateral thigh elicited thermal hypersensitivity for at least 4 h. In this study, we dissected the underlying muscle in addition to incising the skin to prolong this interval. With this added step thermal hypersensitivity lasted 8 h but was similar to baseline for all groups by 24 h, meaning we could not assess the effectiveness of each treatment beyond 8 h. We also have to take into consideration that only thermal hypersensitivity was tested. During a pilot study, mechanical hypersensitivity (von Frey monofilament) was deemed to be impractical due to the animal’s size and anatomy and was therefore not performed.

Bup HCl is widely considered a cornerstone of effective postoperative analgesia and has been used extensively in both laboratory and companion animals for decades. Though Bup HCl does have a longer duration of action than other opioids such as morphine, it still requires administration every 6–12 h to maintain effective plasma concentrations in adult animals [17]. In human pediatric patients aged 6 months to 6 years, Bup HCl given at a dose of 1.5 mcg/kg conferred analgesia for an average of 4.9 h [28]. This duration is likely longer in newborns given the reduced clearance secondary to the immaturity of the glucuronidation system, though the exact duration of action in this population is unknown [29]. More recently, Bup SR became commercially available and confers analgesia for 48–72 h in adult rats, thereby reducing animal handling and subsequent stress [20,21]. Despite the widespread use of Bup SR in adult laboratory animals, no publications exist regarding its safety or efficacy in neonatal animals. Previous studies in adult Sprague-Dawley rats have found that Bup SR administered anywhere between 0.3 to 1.2 mg/kg provides effective antinociception in an incisional pain model for at least 48 h [20]. We chose our high-SR dose (1 mg/kg) based on these results. The low-SR dose (0.5 mg/kg) was based on equivalent dosing with Bup HCl at 0.05 mg/kg every 8 h for 3 d. In the current study, both the low- and high-SR groups effectively attenuated thermal hypersensitivity in neonatal animals for at least 8 h. Surprisingly, analgesic effects of Bup HCl at 0.05 mg/kg in neonatal rats were not observed at 4 h despite previous findings that this same dose provides adequate attenuation of hypersensitivity for 12 h in the incisional pain model in adult rats [26].

Though generally accepted to have a wide margin of safety [30], Bup HCl has been reported to produce dose-dependent respiratory depression and sedation [31], decreased GI transit time [32], and pica behavior [17] in a variety of species. Neonatal procedures are further complicated by the possibility of cannibalism or neglect by the dam postoperatively [19]. We did not note any observable clinical effects associated with buprenorphine, nor were there complications associated with maternal acceptance as typified by the presence of a milk spot and increased weight gain at all time points. Additionally, previous studies evaluating Bup SR have reported skin lesions, scabbing, and ulcerations at the injection site [15,33]. In our current study, there was no visible erythema, scabbing, or irritation of the skin at the administration site for Bup SR in any of the rat pups. We did find that the skin temperature of both the ipsilateral and contralateral thighs was significantly lower in the high-SR treatment group at 1 h as compared to baseline skin temperatures. This is contradictory to studies in adult animals which showed an association between buprenorphine administration and elevated body temperature [34,35]. One potential explanation of our finding is that buprenorphine’s activation of peripheral μ-opioid receptors causes a receptor-mediated release of histamine [36], producing peripheral vasodilation [37] and the potential for subsequent heat loss. These vasodilatory sequelae are likely more pronounced in neonates due to their decreased insulation and limited ability to thermoregulate as compared to adult animals.

From a practical standpoint, the cost of a 5mL vial of 0.5 mg/mL Bup SR from ZooPharm is more expensive than an equivalent volume of Bup HCl ($105 and $75, respectively). However, the volume necessary to singly dose a neonatal rat with either drug was negligible (0.01 mL for Bup and low-SR; 0.02 mL for high-SR). Bup SR is viscous and generally larger gauge needles are recommended for administration. Due to the small administration volumes needed in this study, insulin syringes were used to ensure accurate dosing. Drawing up Bup SR through the irremovable 31-gauge insulin needle did take marginally longer than did pulling up Bup but is very doable.

Based on the results of our current study, a single dose of Bup SR at 0.5 or 1 mg/kg SC provides at least 8 h of postoperative attenuation of thermal hypersensitivity in a neonatal rat incisional pain model. A model causing longer-term pain in neonates is needed to determine the actual length and dose dependency of Bup SR’s efficacy in this demographic. Additional studies measuring the plasma concentrations of Bup SR in neonates, as well as further research examining its effectiveness in different neonatal surgical pain models and with other neonatal laboratory animal species are warranted.
